# IgG4-Related Disease With Lung and Kidney Involvement: A Case Report

**DOI:** 10.7759/cureus.99944

**Published:** 2025-12-23

**Authors:** Andreia Sá Lima, Tiago Castro Pinto, Luísa Veiga de Sousa, P. Ricardo Pereira

**Affiliations:** 1 Internal Medicine, Hospital Pedro Hispano, Unidade Local de Saúde de Matosinhos, Matosinhos, PRT

**Keywords:** autoimmune diseases, fibrosis, immunoglobulin g4-related disease, interstitial nephritis, organizing pneumonia

## Abstract

IgG4-related disease (IgG4-RD) is a rare systemic fibroinflammatory condition that often mimics malignancy, infection, or autoimmune diseases, leading to diagnostic delays. Pulmonary and renal involvement in IgG4-RD are rare but pose significant diagnostic challenges. We describe the case of a man in his early 70s who was admitted due to presumed pneumonia and acute kidney injury (AKI). Despite antibiotic therapy, imaging tests revealed progressive pulmonary consolidations and ground-glass opacities. Laboratory tests showed elevated IgG4, hypocomplementemia, and elevated antinuclear antibody titers. Microbiological and cytological studies of bronchoalveolar lavage fluid were unrevealing. Lung biopsy demonstrated IgG4-rich lymphoplasmacytic infiltrates, confirming IgG4-RD. Months later, worsening kidney function led to the diagnosis of IgG4-related tubulointerstitial nephritis. Initially responsive to corticosteroids, the patient relapsed upon tapering. Rituximab was then introduced, achieving disease remission and sustained stability. This case emphasizes IgG4-RD as a “great masquerader,” highlighting the need for careful integration of clinical, serological, and histological findings for accurate diagnosis.

## Introduction

IgG4-related disease (IgG4-RD) is a rare systemic fibroinflammatory condition that can affect virtually any organ [1,2]. Over the years, distinct disease phenotypes with varying clinical characteristics and prognostic implications have been identified. These include the pancreatobiliary phenotype; the retroperitoneal phenotype, marked by retroperitoneal fibrosis; the head and neck-limited phenotype; and the systemic phenotype, which predominantly affects the salivary glands, pancreas, kidneys, and lungs [1,3].

This disease poses significant challenges in both diagnosis and treatment. First, it has only been widely recognized in the last two decades, with its first formal description in 2003, meaning awareness remains low. Additionally, its multifaceted presentation often mimics neoplastic, infectious, or other immune-mediated diseases, making differential diagnosis particularly challenging [1,3,4]. For many years, the therapeutic management of IgG4-RD was uncertain and relied primarily on expert opinion and small retrospective studies. More recently, clinical trial data have demonstrated the efficacy of B-cell-targeted therapies, marking a shift toward evidence-based treatment strategies [2,5].

Here, we describe a clinical report of IgG4-RD, consistent with the systemic phenotype, involving the lungs, kidneys, and salivary glands. This case highlights the difficult differential diagnosis, as IgG4-RD continues to live up to its reputation as the “great masquerader.”

This article was previously presented as a meeting abstract at the 30º Congresso Nacional de Medicina Interna on May 24, 2024.

## Case presentation

We describe the case of a 73-year-old male with a past medical history of heart failure related to ischemic and valvular heart disease, atrial fibrillation, and dyslipidemia. He was previously admitted with mild hypoxemic respiratory failure (PaO₂/FiO₂ ratio: 270) and documented fever (38.8°C) secondary to community-acquired pneumonia caused by concomitant *Legionella pneumophila* and SARS-CoV-2 infection. Chest CT displayed consolidations in the right upper and middle lobes, along with mediastinal lymphadenopathy. The clinical course was favorable following a 10-day course of levofloxacin and an eight-day course of remdesivir. At discharge, complete resolution of symptoms, normalization of oxygenation, and marked improvement in inflammatory markers supported the working diagnosis of post-infectious radiological changes. A follow-up plan was established, including repeat chest CT and pulmonary function testing (PFT). Two months later, imaging findings were unchanged, and PFT revealed a normal spirometry flow-volume curve and decreased diffusion capacity, raising suspicion for interstitial lung disease (ILD). Further evaluation was not possible, as the patient was lost to follow-up.

Six months later, he was readmitted with a presumptive diagnosis of pneumonia. At this time, he presented with a two-day history of fever, productive cough, and pleuritic chest pain. No additional symptoms were reported, including sinus congestion, skin rash, joint pain, neuropathic symptoms, or hemoptysis. On admission, he was afebrile (36.7°C), with adequate oxygen saturation (SpO₂: 94%) and no signs of respiratory distress. There were bibasilar crackles on lung auscultation. The physical examination was otherwise unremarkable.

Laboratory tests indicated elevated inflammatory markers (white blood cell count and C-reactive protein) and acute kidney injury (AKI) with a creatinine level of 2.0 mg/dL and an estimated glomerular filtration rate (eGFR) of 33 mL/minute on admission (baseline creatinine of 1.0-1.2 mg/dL and eGFR of 60-70 mL/minute). A chest CT demonstrated persistent consolidations in the right upper and middle lobes with associated mediastinal lymphadenopathy, previously documented on prior admission, as well as new centrilobular nodules and ground-glass opacities in the left lower lobe. Antimicrobial treatment with amoxicillin/clavulanate was initiated for *Haemophilus influenzae* non-type B isolated from sputum culture.

Despite this, the patient developed progressive exertional dyspnea and orthopnea, ultimately presenting with moderate hypoxemic respiratory failure (PaO₂/FiO₂ ratio: 196). Following adjustment of diuretic therapy for chronic decompensated heart failure and switch to piperacillin/tazobactam, the patient demonstrated clinical improvement and successfully completed a 14-day course of antibiotics.

**Figure 1:**
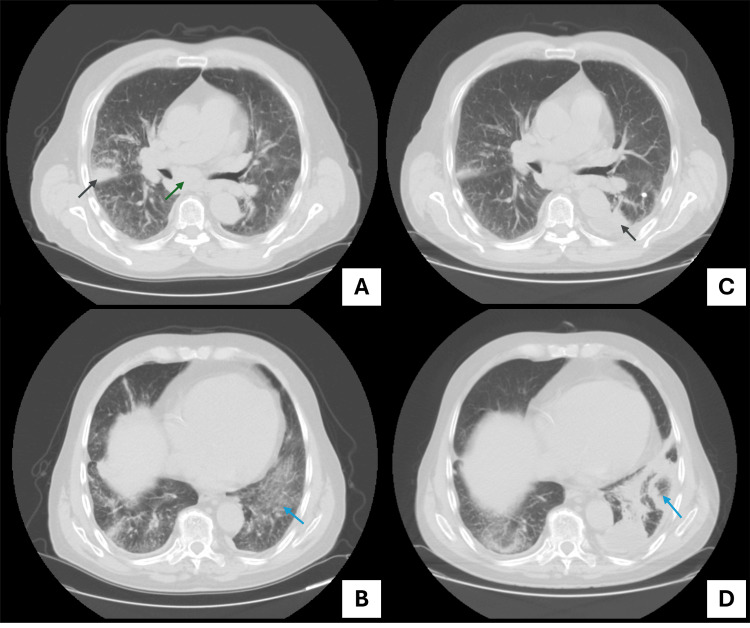
Evolution of radiologic findings on CT scan. Single-level views from the follow-up CT scan performed after the first hospitalization (A and B) and the CT scan obtained during the second hospitalization (C and D). (A) Focal lung consolidation (dark blue arrow) and enlarged mediastinal lymphadenopathy (green arrow). (B) Bilateral ground-glass opacities, more pronounced in the left lower lobe (light blue arrow). (C) Findings similar to those observed in Panel A, with the addition of a new left lower lobe consolidation (dark blue arrow). (D) A left lower lobe consolidation accompanied by fibrotic bands and a radiologic atoll sign, defined as a central focus of ground-glass opacity surrounded by a peripheral ring of consolidation (light blue arrow).

Given the delayed clinical response to antibiotic therapy and persistently elevated inflammatory markers, a repeat chest CT scan was performed on day 12 of admission, revealing clear radiologic progression. Findings included persistent consolidations in the right upper and middle lobes, a new consolidation in the left lower lobe, fibrotic bands, atoll sign, extensive ground-glass opacities, and mediastinal lymphadenopathy (Figure 1).

Additional investigations were conducted to determine the etiology of the pulmonary disease (Table 1). Laboratory findings indicated elevated total IgG levels (3,216 mg/dL), low C4 levels, and an elevated antinuclear antibody (ANA) titer (1:640) with a nuclear homogeneous pattern, with positivity for anti-dsDNA on enzyme-linked immunosorbent assay (ELISA) but negative on immunofluorescence. A comprehensive autoimmune panel, including antibodies associated with systemic lupus erythematosus, Sjögren’s syndrome, systemic sclerosis, and systemic vasculitis, was negative. Urinalysis was normal, with no evidence of hematuria, elevated white blood cells, or proteinuria. Bronchoscopy with bronchoalveolar lavage, bronchial biopsy, and subcarinal lymph node transbronchial biopsy revealed no malignancy, granulomas, or infection. Given these inconclusive results, a transthoracic lung biopsy was performed on the left lower lobe. Histological findings included extensive necrosis, fibrosis, thickened vascular walls, and a dense lymphoplasmacytic inflammatory infiltrate. Ziehl-Neelsen staining was negative, and no granulomas were observed.

**Table 1:** Key Analytical Results from the Second Hospitalization. ^§^: Results became available only on follow-up; ^**^: Microbiological workup includes extensive cultural, serologic, and molecular testing for bacteria and viruses. CRP = C-reactive protein; ESR = erythrocyte sedimentation rate; eGFR = estimated glomerular filtration rate (estimated by CKD-EPI creatinine); WBC = white blood cells; ACE = angiotensin-converting enzyme; SPEP = serum protein electrophoresis; sFLC = serum free light chains; IF = immunofluorescent technique; Anti-GBM = anti-glomerular basement membrane antibody; RF = rheumatoid factor; BS = bronchial secretions; BAL = bronchoalveolar lavage

Analytical workup	D0	D4	D8	D12	D16	D20
	Amoxicillin/clavulanate					
		Piperacillin/Tazobactam				
			Hydroxychloroquine			
CRP (<5.0 mg/L)	185.7	280.3	211.4	103.8	66.3	34.3
ESR (0–20 mm/hour)	24	-	-	-	-	-
Creatinine (0.7–1.3 mg/dL)	2.0	2.0	1.2	1.4	1.9	1.6
eGFR (mL/minute/1.73m^2^)	33	33	57	49	35	41
Hemoglobin (13.0–18.0 g/dL)	15.1	12.8	12.0	12.0	13.2	12.8
WBC (4,000–11,000/µL)	12,050	10,210	7,660	8,600	9,620	7,750
Platelets (150,000–400,000/µL)	123,000	154,000	225,000	270,000	337,000	313,000
Ionized calcium (1.15–1.35 mmol/L)	1.19	-	1.15	-	-	-
ACE (8.0–52.0)	-	-	25.9	-	-	-
SPEP + sFLC	-	Ø M protein	-	-	-	-
IgG (540–1,922 mg/dL)	-	2723.0	-	-	-	-
IgA (101–645 mg/dL)	-	211.0	-	-	-	-
IgM (22–240 mg/dL)	-	49.0	-	-	-	-
C3 (82–195 mg/dL)	-	99.0	-	-	-	-
C4 (15–53 mg/dL)	-	4.9	-	-	-	-
ANA	-	1:640	-	-	-	-
Anti-dsDNA (<10.0 UI/mL ELISA or <1/10 IF)	-	21.0 (ELISA)	-	Negative^§^ (IF)	-	-
Anti-PR3 (<2.0 UI/mL)	-	<0.2	-	-	-	-
Anti-MPO (<5.0 UI/mL)	-	0.2	-	-	-	-
Anti-GBM (<7.0 UI/mL)	-	<0.8	-	-	-	-
RF (<30.0 UI/mL)	-	81.3	-	-	-	-
Cryoglobulins	-	-	-	-	-	Negative^§^
Urinalysis	Normal	-	-	-	-	-
Microbiological workup**	BS: *H. influenza*	-	-	BAL: negative	-	-
Tuberculosis testing	-	BS: negative	-	BAL: negative	-	-

Several differential diagnoses were considered. However, none fully accounted for the clinical presentation. Infectious causes, such as tuberculosis, were excluded based on negative microbiological studies. Neoplastic diseases, including primary lung cancer and lymphoproliferative disorders, were deemed unlikely given imaging findings and biopsy results. ILDs were the focus of the differential.

Among exposure-related ILDs, no occupational or environmental triggers were identified, and recent infection was the only potential contributor to a post-infectious ILD. Connective tissue disease-related ILDs (CTD-ILDs) were considered, given the presence of elevated ANA titers, elevated rheumatoid factor, positive anti-dsDNA (ELISA), and low C4 levels; however, no other clinical features supported the diagnosis of a specific CTD. Among idiopathic ILDs, the radiologic pattern resembled cryptogenic organizing pneumonia, but this is a diagnosis of exclusion and could not be established at that stage. Sarcoidosis was also considered to explain the mediastinal lymphadenopathy, but no granulomas were identified on histopathological evaluation. Systemic vasculitis, such as antineutrophil cytoplasmic antibody (ANCA)-mediated, were only briefly considered, but quickly ruled out, as the clinical presentation was not compatible: renal failure was present with bland rather than active urinary sediment, there were no signs of diffuse alveolar hemorrhage, and ANCAs tested negative.

Although the patient did not meet the criteria for systemic lupus erythematosus, the presence of elevated ANA titers, low C4 levels, and positive anti-dsDNA by ELISA raised suspicion. Hydroxychloroquine was initiated as a relatively safe immunomodulatory agent and used as a bridging therapy during the diagnostic evaluation. Retrospectively, we recognize it as a limitation of this case, as there is no evidence supporting its efficacy in pulmonary manifestations of CTD.

During hospitalization, the patient experienced partial clinical improvement upon completing antibiotic therapy and optimizing heart failure management and was discharged for outpatient follow-up. Continued monthly outpatient follow-up revealed persistently elevated total serum IgG levels, without evidence of monoclonality but with significantly increased serum IgG4 levels. Endobronchial ultrasound-guided biopsy of a mediastinal lymph node showed polyclonal plasmacytosis (9.6%). Upon reviewing the lung biopsy, IgG4-positive plasma cell infiltration was confirmed, solidifying the diagnosis of IgG4-RD with pulmonary involvement.

**Figure 2:**
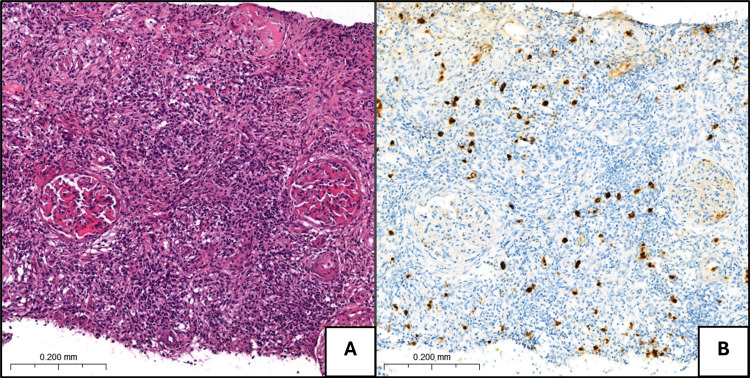
Kidney biopsy. (A) Chronic interstitial nephritis with a dense lymphoplasmacytic inflammatory infiltrate, disruption of the tubulointerstitial compartment, and storiform fibrosis (hematoxylin and eosin, 25×). (B) Increased number of IgG4+ plasma cells (immunohistochemistry, 25×).

Following diagnosis, the patient remained clinically stable, with no evidence of respiratory failure, no worsening renal function, or new organ involvement. Given the need for further evaluation of systemic involvement, a cautious decision was made to withhold corticosteroid therapy while maintaining close follow-up. Three months later, he was hospitalized due to acute kidney failure (eGFR: 16 mL/minute/1.73m²). Despite discontinuing potential nephrotoxic drugs and providing intravenous fluids, no significant improvement in renal function was observed. Urinalysis was normal, and urinary tract obstruction was excluded. A kidney biopsy revealed chronic interstitial nephritis, characterized by a dense lymphoplasmacytic infiltrate (with IgG4-positive plasma cells) and storiform fibrosis (Figure 2). Concurrently, a salivary gland biopsy showed an increased number of IgG4-positive plasma cells, further confirming the systemic nature of the disease.

When kidney involvement became apparent, high-dose corticosteroids (prednisolone 1 mg/kg/day) were initiated, resulting in rapid clinical and biochemical improvement. After three weeks, an initial tapering scheme of 10 mg reduction at two-week intervals was started. However, while on prednisolone 40 mg/day, the patient’s renal function began to decline. Rituximab was then initiated (two infusions given two weeks apart) to induce remission. Following rituximab, corticosteroids were tapered at two-week intervals over a 12-week period until a maintenance dose of 5 mg/day was reached. The patient has remained stable on this regimen for over a year, with improved respiratory symptoms, resolution of pulmonary infiltrates, normalization of serum IgG4 levels, and stable renal function (Table 2).

**Table 2:** Treatment map and analytical overview after diagnosis. **: Failure to taper dose. HCQ = hydroxychloroquine; CRP = C-reactive protein; eGFR = estimated glomerular filtration rate (estimated by CKD-EPI creatinine)

Analytical workup	M0	M3	M4	M6	M9	M12	M24
	HCQ						
		Corticosteroids					
		1 mg/kg	40–50 mg**		Tapered to low dose		
				Rituximab			
				1 g (2 infusions)			
CRP (<5.0 mg/L)	5.7	60.4	1.0	14.0	12.3	10.3	2.4
Creatinine (0.7–1.3 mg/dL)	1.9	3.5	1.9	2.7	2.0	1.9	1.6
eGFR (mL/minute/1.73m^2^)	35	16	35	22	32	33	41
IgG1 (240–1,118 mg/dL)	2,390.0	-	-	-	476.0	-	590.0
IgG4 (7–89 mg/dL)	762.0	-	-	-	32.7	-	63.8

## Discussion

IgG4-RD is an autoimmune condition characterized by fibroinflammatory lesions, typically featuring a dense lymphoplasmacytic infiltrate rich in IgG4-positive plasma cells [3,6]. The literature describes this disease as a continuum between proliferative and fibrotic injury. However, it remains unclear whether these are distinct disease phenotypes or simply different stages of its natural progression [6,7]. In the proliferative stage, self-antigens trigger immune activation, expansion of pathogenic B and T-cell populations, and infiltration of tissues by plasmablasts and plasma cells [6]. In contrast, the fibrotic stage is characterized by immune cell-driven secretion of pro-fibrotic factors, leading to fibroblast activation and stromal proliferation [7]. These stages can be distinguished by clinical presentation and treatment response. The proliferative phenotype is typically centered on glandular and epithelial tissues, whereas the fibrotic phenotype primarily affects extraglandular sites, such as the retroperitoneum. Moreover, the proliferative stage is often associated with higher serum IgG1 and IgG4 levels and hypocomplementemia, as described in the case above [6]. Regarding treatment, patients in the proliferative or early fibrotic stages generally respond better to therapy [6,7].

Pulmonary involvement in IgG4-RD

Several reports have described IgG4-RD with pulmonary involvement, often manifesting as a mass/pseudotumor, interstitial pneumonitis, or organizing pneumonia [8]. Clinical features are frequently nonspecific, ranging from asymptomatic disease to common symptoms such as cough, dyspnea, chest pain, and hemoptysis. Radiologic findings vary widely, including pleural thickening, alveolar-interstitial infiltration, broncho-vascular bundle thickening, mediastinal lymphadenopathy, ground-glass opacities, nodules, and cavitations [9,10].

In this case, clinical symptoms were masked by concurrent bacterial lung infection and acute heart failure, which contributed to the delayed diagnosis. Regarding radiologic findings, this case exhibited features suggestive of interstitial pneumonitis (e.g., ground-glass opacities) and organizing pneumonia (e.g., fibrotic bands, atoll sign). It was precisely these radiologic abnormalities that prompted a broader investigation into alternative etiologies of pulmonary involvement.

Ultimately, persistently elevated IgG levels provided a key diagnostic clue. Once suspected, the diagnosis was quickly confirmed by elevated serum IgG4, an abundance of plasmocytes in the mediastinal adenopathy, and histopathological findings, including storiform fibrosis and IgG4-positive plasma cells. However, of note, serum IgG4 is not a highly sensitive biomarker for this condition, as only 55% of patients with IgG4-RD exhibit elevated levels [2,3].

Kidney involvement in IgG4-RD

IgG4-related kidney disease (IgG4-RKD) most commonly presents as tubulointerstitial nephritis, though a spectrum of renal manifestations has been reported, including membranous glomerulonephritis, renal masses, and retroperitoneal fibrosis. The latter can lead to hydronephrosis and post-renal injury [3,11]. IgG4-RKD is often asymptomatic or mildly symptomatic, typically identified through elevated serum creatinine or proteinuria on routine testing. However, severe cases may present with AKI and, in rare instances, progress to end-stage renal disease [11]. The extent of fibrosis is a key predictor of long-term renal outcomes [6,11].

In this case, the patient developed AKI, prompting a broad differential diagnosis. Given his polypharmacy, nephrotoxic injury was initially considered the most likely cause. However, a renal biopsy confirmed IgG4-RKD, characterized by storiform fibrosis and a dense interstitial lymphoplasmacytic infiltrate rich in IgG4-positive cells, hallmark features of IgG4-RD [4,6,11]. Unlike drug-induced interstitial nephritis, which typically presents with an active urinary sediment, IgG4-RKD exhibits a bland sediment [11]. Ultimately, integration of clinical, serological, and histological findings established the definitive diagnosis.

Management of IgG4-RD

This case can be classified within the proliferative/inflammatory stage of IgG4-RD, typically associated with a favorable response to treatment [6]. Glucocorticoids are considered the first-line therapy for inducing remission in IgG4-RD [1]. The patient responded well to glucocorticoids, with reduced inflammation, improved kidney function, and resolved pulmonary symptoms.

In retrospect, earlier initiation of corticosteroid therapy might have mitigated renal involvement, and this case highlights the importance of timely treatment in IgG4-RD despite initial clinical stability. Although IgG4-RD often follows a slowly progressive course and urgent treatment is not universally required, current recommendations support early initiation of therapy in symptomatic patients and in asymptomatic patients with evidence of progressive disease, including the lungs and kidneys [1]. In contrast, observation with close monitoring may be appropriate for individuals with asymptomatic, limited disease and no evidence of progression, which was not applicable in this case.

Due to the relapsing nature of the disease, evidenced by deterioration in kidney function upon glucocorticoid tapering, and significant side effects of high-dose glucocorticoids, a second-line treatment had to be considered.

B-cell-targeted therapies have emerged as effective alternatives [1]. Beyond targeting the proliferative stage, B-cell depletion also influences the fibrotic stage, as a subset of B cells is involved in the secretion of pro-fibrotic factors and fibroblast activation [3,12]. Evidence supporting their efficacy has historically come from retrospective and prospective cohort studies [1]. A 2021 meta-analysis demonstrated high remission rates using rituximab in IgG4-RD, although controlled trials were lacking at that time [13].

More recently, the MITIGATE Trial (inebilizumab for IgG4‑RD) has provided prospective, randomized controlled trial evidence establishing the efficacy of B‑cell-targeted therapy. Patients treated with inebilizumab, an anti-CD19 B-cell depleting antibody, while on an identical glucocorticoid taper regimen, achieved significantly higher rates of sustained remission, experienced fewer relapses, and reported a lower incidence of steroid-related adverse events [5]. At the time this case occurred, inebilizumab was not yet approved. Nonetheless, it exemplifies the successful use of rituximab in real-world practice.

After remission, long-term maintenance therapy must be carefully considered. Given the organ-threatening manifestations in this case, the patient was maintained on low-dose glucocorticoids (5 mg/day). While this strategy is widely employed, B-cell-targeted therapies and, less frequently, conventional immunosuppressants, have also demonstrated clinical benefit in maintenance therapy [1,5].

Finally, novel therapies are under investigation that may further reshape the therapeutic landscape. These include cytokine-targeting agents, such as tofacitinib and baricitinib, and next-generation B-cell-targeted therapies, such as obexelimab, which has shown promising results in a recent phase 2 trial [14].

## Conclusions

IgG4-related disease is an emerging systemic condition with a broad and evolving clinical spectrum, requiring clinicians to maintain vigilance for atypical or multi-organ presentations. Clinical, serological, and histologic features are all essential in defining an IgG4-RD diagnosis, with histopathology serving as the diagnostic gold standard. Early initiation of glucocorticoids remains the cornerstone of therapy. However, steroid-sparing agents such as rituximab or inebilizumab may be considered in cases of an inadequate induction response or steroid dependence.
